# Bitter gourd (*Momordica charantia*) possess developmental toxicity as revealed by screening the seeds and fruit extracts in zebrafish embryos

**DOI:** 10.1186/s12906-019-2599-0

**Published:** 2019-07-24

**Authors:** Muhammad Farooq Khan, Nael Abutaha, Fahd A. Nasr, Ali S. Alqahtani, Omar M. Noman, Mohammad A. M. Wadaan

**Affiliations:** 1 Bioproducts Research Chair, College of Science, Department of Zoology, King Saud University, P.O. Box 2455, Riyadh, 11451 Kingdom of Saudi Arabia; 2 Medicinal Aromatic, and Poisonous Plants Research Center, College of Pharmacy, King Saud University, Riyadh, 11451 Saudi Arabia

**Keywords:** *Momordica charantia*, Cardiac hypertrophy, *Danio rerio*, Developmental toxicity

## Abstract

**Background:**

Bitter gourd (*Momordica charantia*) has attracted the focus of researchers owing to its excellent anti-diabetic action. The beneficial effect of *Momordica charantia* on heart has been reported by in vitro and in vivo studies. However the developmental toxicity or potential risk of *M. charantia* on fetus heart development is largely unknown. Hence this study was designed to find out the developmental toxicity of *M. charantia* using zebrafish (*Danio rerio*) embryos.

**Methods:**

The crude extracts were prepared from fruit and seeds of *M. charantia.* The Zebrafish embryos were exposed to serial dilution of each of the crude extract. The biologically active fractions were fractionated by C18 column using high pressure liquid chromatography. Fourier-transform infrared spectroscopy and gas chromatography coupled with mass spectrophotometry was done to identify chemical constituents in fruit and seed extract of *M. charantia*.

**Results:**

The seed extract of *M. charantia* was lethal with LD_50_ values of 50 μg/ml to zebrafish embryos and multiple anomalies were observed in zebrafish embryos at sub-lethal concentration. However, the fruit extract was much safe and exposing the zebrafish embryos even to 200 μg/ml did not result any lethality. The fruit extract induced severe cardiac hypertrophy in treated embryos. The time window treatment showed that *M. charantia* perturbed the cardiac myoblast specification process in treated zebrafish embryos. The Fourier-transform infrared spectroscopy analyses revealed diverse chemical group in the active fruit fraction and five new type of compounds were identified in the crude seeds extract of *M. charantia* by gas chromatography and mass spectrophotometry.

**Conclusion:**

The teratogenicity of seeds extract and cardiac toxicity by the fruit extract of *M. charantia* warned that the supplementation made from the fruit and seeds of *M. charantia* should be used with much care in pregnant diabetic patients to avoid possible damage to developing fetus.

**Electronic supplementary material:**

The online version of this article (10.1186/s12906-019-2599-0) contains supplementary material, which is available to authorized users.

## Background

Herbs have been used in traditional medicine for centuries to cure various ailments, including diabetes mellitus. Bitter gourd is a vegetable widely used in East Asian, South Asian, and Southeast Asian cuisines, and its fruit is generally consumed as a cooked food in its green or early yellowing stage". Moreover, bitter gourd has long been used as an herbal medicine in Asian and African traditional medicines [1–3]. One of the most common uses of *M. charantia* is as an antidiabetic agent. The application of *M. charantia* to treat diabetic mellitus has been extensively studied, which is apparent by the increasing number of publications over the years. PubMed and web of science searches using the key word “*Momordica charantia”* resulted in 1253 articles for the last 10 years only (2009–2018). Quite recently, several clinical trials have been conducted to examine the efficacy of *M. charantia* in diabetic patients, and *M. charantia* supplementation was shown to be quite successful in lowering elevated fasting plasma glucose level in prediabetes patients [4–7]. The exact mechanisms of the antidiabetic and anti-obesity effects of *M. charantia* are unknown; however, some of the active components isolated from *M. charantia* are thought to be structurally similar to human insulin [8]. Besides being a potent anti-diabetic supplementation, various parts of *M. charantia* have also been used as a medicine to cure various conditions, such as infection [9–13], wounds [14–16], and osteoarthritis [4, 17]. The plant are also used as laxative, contraceptive, abortifacient, and anthelmintic agents, and also to treat various conditions, such as scabies, jaundice, pneumonia, dysmenorrhea, eczema, gout, leprosy, piles, psoriasis, and rheumatism [18–21]. *M. charantia* has also been shown to possess strong anticancer properties and strong cytotoxicity in various human cancer cell lines, such as lymphoid leukemia [22], lymphoma, melanoma [23], breast cancer [24], prostatic cancer [25–28], squamous carcinoma of the tongue and larynx. Owing to the promising biological properties and medicinal use of this plant, more than 200 compounds have been isolated and identified from the fruit, leaves, vines, seeds, and roots of *M. charantia* [29, 30].

Cardiomyopathy is one of the most common complications of diabetes mellitus which is believed to be due to the oxidative damage of the heart tissues. The antioxidant property of *M. charantia* has been proven by various studies [31–36]. The beneficial effect of *M. charantia* against the oxidative stress complications in the heart of diabetic rats was demonstrated by [37]. Moreover, the myocardial protective effect from polysaccharides of *M. charantia* has been observed in an isoproterenol (ISP) induced myocardial infarction rat model [38]. *M. charantia* fruit extract have also showed cardio-protective action in the treatment of diabetic cardiac fibrosis in streptozotocin (STZ) induced diabetes in Sprague-Dawley rats by lowing the expression of type III, type IV collagens and Hydroxyproline content which reverted morphological damages in the heart to normal [39]. However, there is no scientific evidence so far, which can elucidate the action of *M. charantia* on fetal heart development either in animal or in human. The international drug regulatory guidelines emphasize that drugs under development that will be administered to pregnant women must be tested for developmental toxicity in suitable animal models [40, 41].

The main objective of this study was to analyze the effect of *M. charantia* on embryonic heart development using zebrafish embryos. Zebrafish *(Danio rerio)* belongs to the teleost family, and zebrafish embryos are routinely used in developmental toxicology testing as an excellent model organism [42–48]. The main advantage of using zebrafish for such toxicological studies is that, as zebrafish embryo develops outside of mother, thus eliminating the effects of mother on the fetus development and hence toxicity of any compound can easily be assessed directly in the embryos. These mother-related effects are misleading to discover the sensitivity and mechanism of the fetus for developmental abnormalities [49].

Methanol extracts was prepared from the fruit and seeds of *M. charantia*. The crude extracts were further fractionated in trichloromethane, ethyl acetate, methanol, and water. The biologically active fraction (which induced significant level of developmental toxicity in zebrafish embryos) was further fractionated by silica gel column chromatography and high-performance liquid chromatography (HPLC). Phenol and flavonoid contents of the active fraction (ethyl acetate fraction) were determined. The active constituent in seed extract were identified by gas chromatography spectroscopy (GC-MS).

## Material and methods

### Materials

All reagents used in this study were of HPLC-grade and purchased from Sigma Aldrich.

### Methods

#### Collection and authentication of plant material

The fruit and the seeds of bitter gourd (*M. charantia*) were obtained from commercial local vegetable market and taxonomical identification was confirmed by Dr. Jacob Thomas Pandalayil, Assistant Professor/Curator, Herbarium (King Saud University) in the Department of Botany College of Science, King Saud University, Riyadh, Kingdom of Saudi Arabia. A specimen voucher sample (Acc. No. (KSU) 10626) was deposited in KSU herbarium.

### Plant extraction

Seedless, fresh fruits (200 g) of *M. charantia* were cut into small pieces and washed with tap water followed by distilled water. The fruits were air-dried in a ventilated area. After drying, the fruits were grounded using a commercial blender. The dried powder was extracted with methanol using a sonicator at 25 °C for 30 min and then kept in a shaking incubator for 24 h at 250 rpm and 30 °C. The extract was centrifuged at 12000 rpm for 10 min. The solvent was then evaporated using a rotary evaporator at 45 °C, and the extract was weighed and kept at − 80 °C until use. Crude seed extract was also prepared in a similar manner.

### Column chromatography

*M. charantia* fruit extract (2 g) was subjected to column chromatography using silica gel 60 silanized (0.063–0.200 mesh; Merck, Darmstadt, Germany). The sample was prepared by adsorbing 2 g of the extract to 20 g of silica and then left to dry. The dry powder was applied on top of the column (5 × 25 cm) and then eluted using trichloromethane, ethyl acetate, and methanol-water (50:50) with pressure. Each solvent at a volume of 500 ml was collected in a beaker.

### C_18_ cartridge extraction

C_18_ cartridges were used to further fractionate the ethyl acetate active fraction isolated from *M. charantia* fruit. The active fraction (2 mL) was diluted with 8 mL of distilled water. The SPE cartridge used was Chromabond C18ec-cartridge (Macherey & Nagel). The cartridge was attached to a vacuum and sequentially conditioned by passing 10 mL of methanol, 10 mL of Milli-Q water, and 10 mL of methanol-water (2:8 v/v). Diluted active fraction (10 mL) was loaded onto the preconditioned cartridge and eluted at a drop-wise to ensure efficient adsorption of the compounds. Elution of C_18_ cartridge-bound compounds was achieved by adding 10 mL of methanol-water (2:8), followed by methanol drop-wise. Finally, two fractions were collected and concentrated using a rotary evaporator at 45 °C. The fractions were dried, reconstituted, and stored at − 80 °C until use.

### Phenol estimation

Total phenolic content of the extracts was measured by the Folin-Ciocalteu method [11]. Briefly, 12.5 μL of extract (1 mg/mL) were mixed thoroughly with 50 μL of distilled water and 12.5 μL of 25% Folin-Ciocalteu reagent for 5 min. Next, 125 μL of 7% (w/v) Na_2_CO_3_ (sodium carbonate) was added to the mixture, which was then allowed to stand for 1.5 h at room temperature (25 ± 2 °C) in the dark. Absorbance was measured at 760 nm using a microplate reader. Total phenol content was quantified using the standard curve of gallic acid (Joshi et al. 2013).

### Flavonoid estimation

Total flavonoid content in the extracts was quantified according to a method by Ghosh et al. (2008). In brief, 100 μL of extract was mixed with 100 μL of 2% aluminum chloride. After 10 min of incubation, absorbance was measured at 368 nm. The standard curve used to estimate total flavonoids was set using quercetin standard solution (100 to 800 mg/ml).

### GC/MS analysis

The gas chromatography-mass spectroscopy (GC-MS) analysis was performed in a Perkin Elmer Clarus 600 gas chromatograph inked to a mass spectrometer essentially same as described previously [50].

### Treatment of zebrafish embryos

The embryos were obtained by natural pairwise breeding. The breeding pairs were set at evening after sunset in 3-l breeding tanks purchased from Pentair **(**Pentair Aquatic Eco-Systems, Inc., Apopka, FL, USA). The embryos were collected the following morning by siphoning after the zebrafish spawned at first sunlight. The eggs were washed with distilled water and transferred to embryo water [NaCl 5.03 mM, KCl 0.17 mM, CaCl2•2H_2_O 0.33 mM, MgSO_4_•7H_2_O 0.33 mM, and methylene blue 0.1% (w/v)]. Synchronized stage embryos at 8 cell stage were exposed to serial dilutions of the extracts in sterile 35-mm glass dishes. Untreated or mock 0.5% (v/v) methanol-treated embryos served as controls. The embryos were treated for up to 6 days (5 dpf). The experiment was repeated at least three times by using different batches of embryos each time. The criteria to confirm the teratogenic effect of *M. charantia* in zebrafish embryonic development was when more than 60% of treated embryos had same effect and also show same p in all three biological replicates.

After the end of experiment the embryos were euthanized by 0.03% Tricaine mesylate (Tricaine methanesulfonate, TMS, MS-222, Cat # E10521,Sigma Aldrich), freeze and discarded as safe biological waste.

### Statistics

Origin (version 6.1052; Origin Lab Corp Northampton, MA, U.S.A.) was used for statistical analysis to calculate the standard deviation between three biological replicates.

## Results

### The fruit and seed extracts of *Momordica charantia* showed different levels of developmental toxicity in zebrafish embryos

To evaluate the developmental toxicity of the fruit and seeds of *M. charantia,* zebrafish embryos were treated with serial dilutions (1 to 400 μg/ml) of crude fruit and seed extracts of *M. charantia*. Zebrafish embryos were exposed to the extracts starting from the eight-cell stage of embryonic development up to 5 dpf. The mortality or teratogenic effects of the extracts in live embryos were recorded at 24, 48, and 72 h of post fertilization (hpf).

### Lethality

The crude seed extract of *M. charantia* was more toxic than the crude fruit extract. The dose response of zebrafish embryos exposed to crude extracts is shown in Table 1. The LC_50_ value of the *M. charantia* seed extract in zebrafish embryos was 50 μg/ml, whereas the fruit extract was found to be harmless, with no significant mortality observed in the zebrafish embryos treated with up to 400 μg/ml.

**Table 1:** Developmental toxicity (lethality) of fruit and seeds extract of *M. charantia* in zebrafish embryos

Concentration μg/ml	% Mortality^a^	
	Fruit extract	Seeds extract
1	0.0 ± 0.00	0.00 ± 0.0
5	0.0 ± 0.00	2.0 ± 0.29
15	0.0 ± 0.00	25 ± 0.58
45	0.0 ± 0.00	40 ± 0.26
135	3.0 ± 0.14	100 ± 0.00
400	10.0 ± 0.61	100 ± 0.00

^a^ Mean of three biological replicates ± Standard deviation

### Seed extract of *Momordica charantia* induced multiple teratological defects in developing zebrafish embryos

**Fig. 1:**
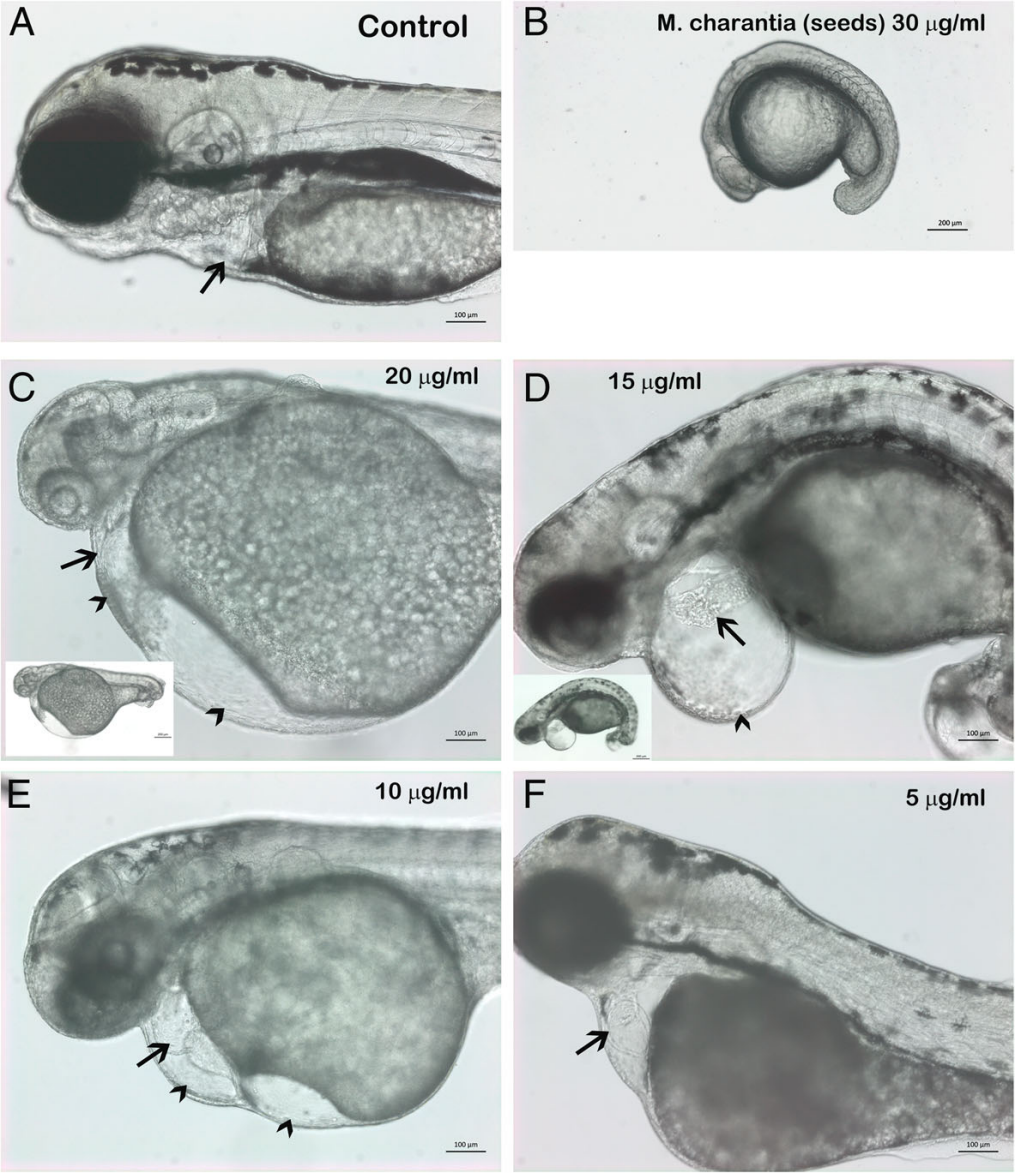
Seed extract of *Momordica charantia* induced multiple teratological defects in developing zebrafish embryos. Representative photomicrograph of live zebrafish embryos at 3dpf control (**a**) and treated with various concentration of seeds extract of *M. charantia* (B-F). The highest concentration in which treated embryos could survive was 50 μg/ml and the representative image of treated embryos are presented in **b**. The embryos treated with 50 μg/ml of seeds extracts were severely developmentally delayed as compared to mock treated embryos as shown in **a**. The control embryos developed to 72hpf, after two days but treated embryos were at 15 somite stage after two days of exposure**. c** The zebrafish embryos which were treated with 25 μg/ml of the seeds extract exhibited multiple teratogenic effect. The treated embryos were developmentally delayed as compared to control, had small head, enlarged yolk (yolk), severe cardiac hypertrophy (arrow), enlarged cardiac cavity (arrow head) absence of tail extension (shown in small inset) absence of circulation (shown in Additional file 1 as supplementary data **d).** The zebrafish embryos treated with 15 μg/ml of seeds extract of *M. charantia* also had mild level of developmental delay, enlarged cardiac cavity (arrow head). The heart was severely affected as both atrium and ventricle were fused representing as string (black arrow). The treated embryos had curved trunk as shown in small at lower magnification. **e.** the zebrafish embryos treated with 10 μg/ml of the seeds extract had mild level of developmental delay but the treated embryos ad severe cardiac hypertrophy (arrow) and also enlarged cardiac cavity (arrow head). **f**). zebrafish embryos treated with 5 μg/ml of the seeds extract did not show development delay however, the cardiac hypertrophy (arrow) was evident in treated embryos. All embryos anterior to the left

The seed extract of *M. charantia* at sub-LC_50_ dose interfered with the development and growth of zebrafish embryos. As shown in Fig. 1, the treated embryos showed significant level of developmental arrest, as compared to the mock (0.5% methanol V/V)-treated or non-treated zebrafish embryos. As it is evident in Fig. 1 and Table 2, the embryos which were treated with seeds extract exhibited gross abnormalities and development arrest on a dose dependent manner. The zebrafish embryos which were treated with 30 μg/ml of the crude extract *M. charantia* prepared from seeds, showed most severe developmental arrest. The treated embryos did not develop after 15 somite stage (16hpf) whereas, the control embryos had attained the protruding mouth stage (~ 72hpf) during the same time period. The embryos which were treated with 20 μg/ml of the seeds extract (Fig. 1c) exhibited enlarged yolk, cardiac hypertrophy with cardiac edema, absence of circulation, absence of tail extensions, and had no pigmentation compared to control embryos. These embryos also failed to hatch. The embryos which were treated with 15 μg/ml crude extract of seeds (Fig. 1d) showed mild level of developmental arrest and but had severe cardiac toxicity. They had cardiac edema and the heart had fused atrium and ventricle resulting a string in the heart and thus resulting poor circulation. The embryos had curved tail and also absence of tail extension. The embryos which were treated with 10 μg/ml of seeds extract (Fig. 1e and Additional file 2) showed mild developmental arrest. The treated embryos had severe cardiac hypertrophy and cardiac edema. The zebrafish embryos which were treated with 5 μg/ml of the seeds extract (Fig. 1f) showed very mild level of development arrest but exhibited severe cardiac hypertrophy.

**Table 2:** Teratogenic dose response of zebrafish embryos exposed to crude seed extract of *M. charantia* at 72hpf

Concentration (μg/ml)	Developmental arrest^a^		Cardiac toxicity^a^		Blood circulation^a^		Hatching^a^	
	Mean	± S.D	Mean	±S.D	Mean	±S.D	Mean	± S.D
Control	0	0	0	0	0	0	0	0
1	0	0	0	0	0	0	0	0
5	0	0	85	0.33	85	0.33	0	0
10	0	0	100	0.00	100	0.00	5	0.67
15	0	0	100	0	100	0	100	0
20	100	0	100	0	100	0	100	0
25	100	0	100	0	100	0	100	0
30	100	0	100	0	100	0	100	0

^a^ Percentage of embryos with developmental defect. The values are the mean of three biological replication ± Standard deviation (S.D)

### GC-MS analysis of phytochemical compounds in *Momordica charantia* seed extract

**Fig. 2:**
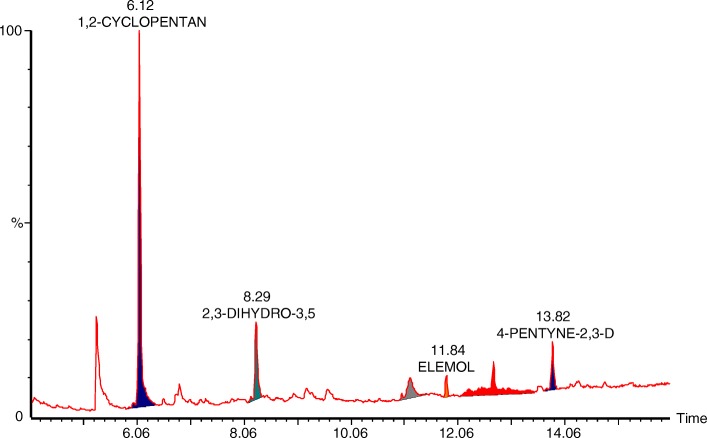
GC-MS analysis of phytochemical compounds in BG seed methanol extract. The highest peak with least retention time was identified as 1,2- CYCLOPENTANEDIONE which is newly identified compound from the seeds of *M.charantia*

The gas chromatography-mass spectroscopy (GC-MS) analysis was performed to identify the active phytochemical present in the seed extract of *M. Chrantia*. Table 3 and Fig. 2 contain the newly identified phytochemical compounds and details GC-MS analysis from the seed extract of *M. charantia.*

**Table 3:** Compounds identified in in BG seed methanol extract using GC-MS

Compound Name	Chemical formula	Molecular weight (g/mol)	RT (min)	Area	Area%
1,2-CYCLOPENTANEDIONE	C_5_H_6_O_2_	98.101	6.11	3319109	59.830
2,3-DIHYDRO-3,5-DIHYDROXY-6-METHYL-4H-PYRAN-4-ONE	C_6_H_8_O_4_	144.126	8.29	1168901	21.070
1,3;2,5-DIMETHYLENE-L-RHAMNITOL	C_8_H_14_O_5_	190.195	11.18	639484	11.530
ELEMOL	C_15_H_26_O	222.372	11.85	75925	1.370
(−)-SELINA-4.ALPHA.,11-DIOL	C_16_H_28_	220.4	12.72	142414	2.570
BETA.-EUDESMOL	C_15_H_26_O	222.372	13.82	201741	3.640

### Crude fruit extract of *Momordica charantia* induced cardiac hypertrophy in treated zebrafish embryos

**Fig. 3:**
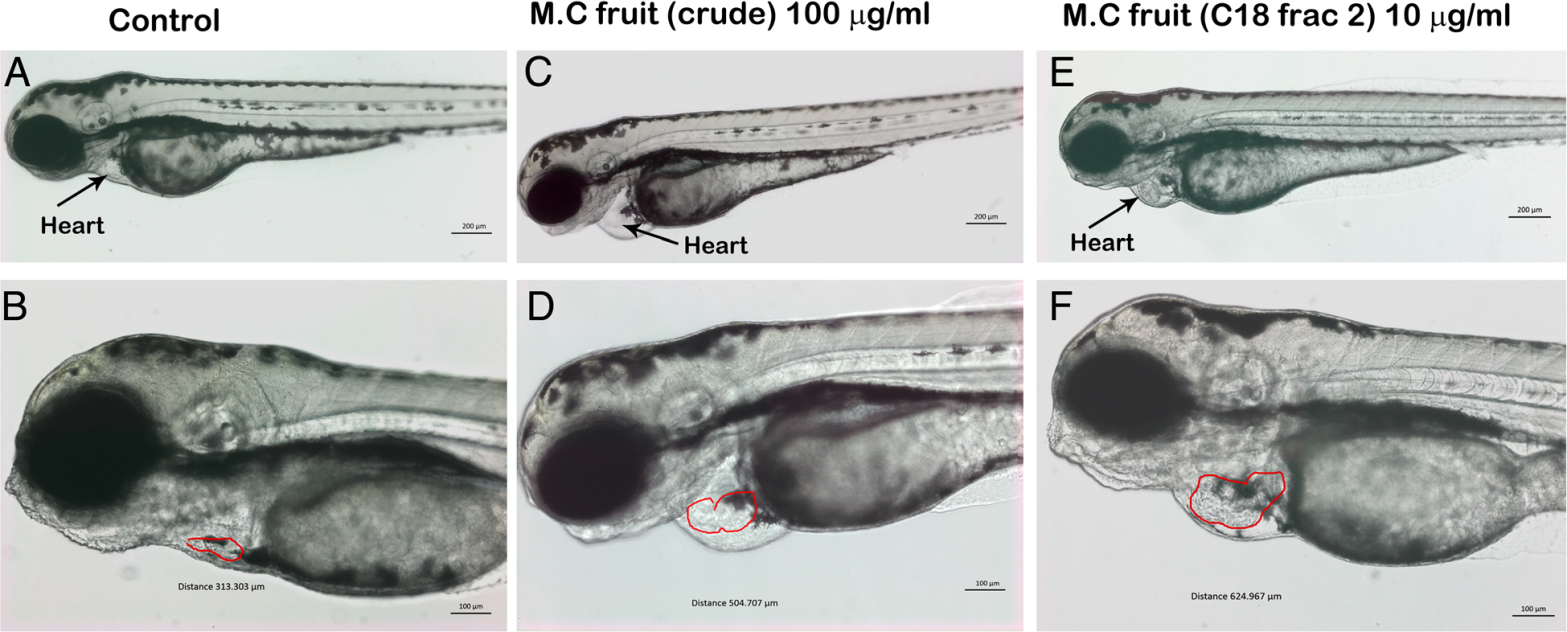
Crude fruit extract of *M. charantia* induced cardiac hypertrophy in treated zebrafish embryos. Representative photomicrograph of live zebrafish embryos at 3dpf. **a** and **b** Mock (methanol 0.5% V/V) exposed embryos showed normal embryonic development and heart growth (black arrow). **c** and **d** the Zebrafish embryos treated with methanol fruit extract of *M. charantia* (100 μg/ml). The treated embryos are not developmentally delayed as the protruding mouth structure which is prominent feature of zebrafish embryos at 72 hpf stage can also be seen clearly in treated embryos (arrow head). However, the treated embryos exhibited enlarged cardiac chamber (edema) and cardiac hypertrophy (arrow head). B and D are the higher magnification showing the heart of the same embryos presented in images A and C. The red encircled areas shows the areas which has been shown in μm at the bottom of the image. **e** and **f)** Zebrafish embryos which were exposed to the fraction 2 (10 μg/ml) obtained from the C18 column fractionation of ethyl acetate fraction of fruit extract of *M. charantia*. The treated embryos did not show developmental delay but had particular cardiac hypertrophy represented by black arrow. The area of the heart is almost 3X times bigger as compared to mock treated control embryos. Scale bar: 50 μm

The crude fruit extract of *M. charantia* was well tolerated by zebrafish embryos and no mortality was observed even at the highest concentration used (200 μg/ml). The zebrafish embryos treated with the methanol fruit extract showed unaltered embryonic development as the treated embryos were at the same developmental stage as the mock (0.1% methanol v/v)-treated counterpart. The cardiac development in control embryos was not affected, and as shown in Fig. 3a and b and Additional file 3, the size of heart was normal with active circulation. However, as evident from Fig. 3c, d, and Table 4 the zebrafish embryos exposed to the crude methanol extract of *M. charantia* developed severe cardiac edema and cardiac hypertrophy. Cardiac hypertrophy was observed in 100% of embryos (*n* = 200 ± 5) which were treated with the crude fruit extract at ≥30 μg/ml. However zebrafish embryos treated with crude fruit extract of *M. charantia* less than 30 μg/ml did not exhibited any noticeable embryonic abnormalities or cardiac toxicity. The measurement of heart size had revealed that the heart of treated embryos were almost double the size as compared to the heart in untreated control embryos (Fig. 3b and d). The area of heart was measured microscopically, from five control and five treated embryos at same resolution and at same developmental stage. The mean area of heart from untreated embryos at 72 hpf was 314 ± 0.70 μm, whereas the mean area of heart was 504.1 ± 0.74 μm of those embryos which were treated with 200 μg/ml of crude fruit extract of *M. charantia*.

**Table 4:** Teratogenic dose response of zebrafish embryos exposed to crude and fraction of fruit extract of *Momordica charanti* at 72hpf

Con. (μg/ml)	Developmental arrest^a^		Cardiac toxicity^a^		Blood circulation^a^		Hatching^a^	
	Mean	± S.D	Mean	± S.D	Mean	± S.D	Mean	± S.D
Fruit crude								
Control	0	0	0	0	0	0	0	0
1	0	0	0	0	0	0	0	0
5	0	0	0	0	0	0	0	0
15	0	0	0	0	0	0	0	0
20	0	0	0	0	0	0	0	0
25	0	0	0	0	0	0	0	0
30	0	0	0	0	0	0	0	0
50	0	0	100	0	100	0	0	0
Fruit C18 frac. #2								
Control	0	0	0	0	0	0	0	0
1	0	0	0	0	0	0	0	0
5	0	0	5	0.25	0	0	0	0
10	0	0	100	0	100	0	0	0
15	0	0	100	0	100	0	0	0

^a^ Percentage of embryos showing the developmental defect. The values are the mean of three biological replication ± Standard deviation (S.D)

The fruit extract of *M. charantia* also affected the heartbeat rate in treated zebrafish embryos significantly. The control embryos (*n* = 150) had a heartbeat rate of approximately 182 ± 5 beats per min (mean heart beat rate from five embryos at 72 hpf), whereas that in *M. charantia*-treated embryos (n = 150) was ~ 90 ± 3 (*p* value 0.0005) beats per min.

The crude extract prepared from the fruit of *M. charantia*, effected only the heart development process without affecting any other organs in treated zebrafish embryos, which mean that the crude fruit extract have a constituent which caused the cardiac toxicity in zebrafish embryos. In order to identify the cardiac toxicity causing component, the fruit crude extract of *M. charantia* was subjected to column chromatography using silica gel. The yields were 140 mg, 80 mg, and 1.60 g for tri- chloromethane, ethyl acetate, and methanol fractions, respectively. The fractions obtained were tested in zebrafish embryos to evaluate their potential toxicity on cardiac development. Among three fractions, only the ethyl acetate fraction induced cardiac hypertrophy in treated zebrafish embryos. This ethyl acetate fraction was further fractionated by HPLC using C_18_ cartridge, which fetched two fractions. The yield of fraction 1 was 36 mg and fraction 2 was 12 mg. These two fractions were also screened in zebrafish embryos in order to see which fraction could reproduce the cardiac toxicity phenotype as was observed with crude fruit extract. Fraction 1 did not produce any noticeable toxicity including the cardiac toxicity in treated zebrafish embryos, however, fraction # 2 successfully replicated the cardiac toxicity phenotype in zebrafish embryos and also at much lower concentration i.e. at 10 μg/ml as compared to that of original crude extract (Fig. 3e and f and Table 4) which indicates that this fraction have higher amount of cardiac toxicity component as compared to crude extract. The heart area measurement revealed that the areas of heart of zebrafish embryos which were exposed to fraction #2 had severe cardiac hypertrophy and heart areas was not only larger than control embryos but also significantly larger than the heart of embryos which were treated with crude fruit extract of *M. charantia* (Fig. 3e and f).

**Fig. 4:**
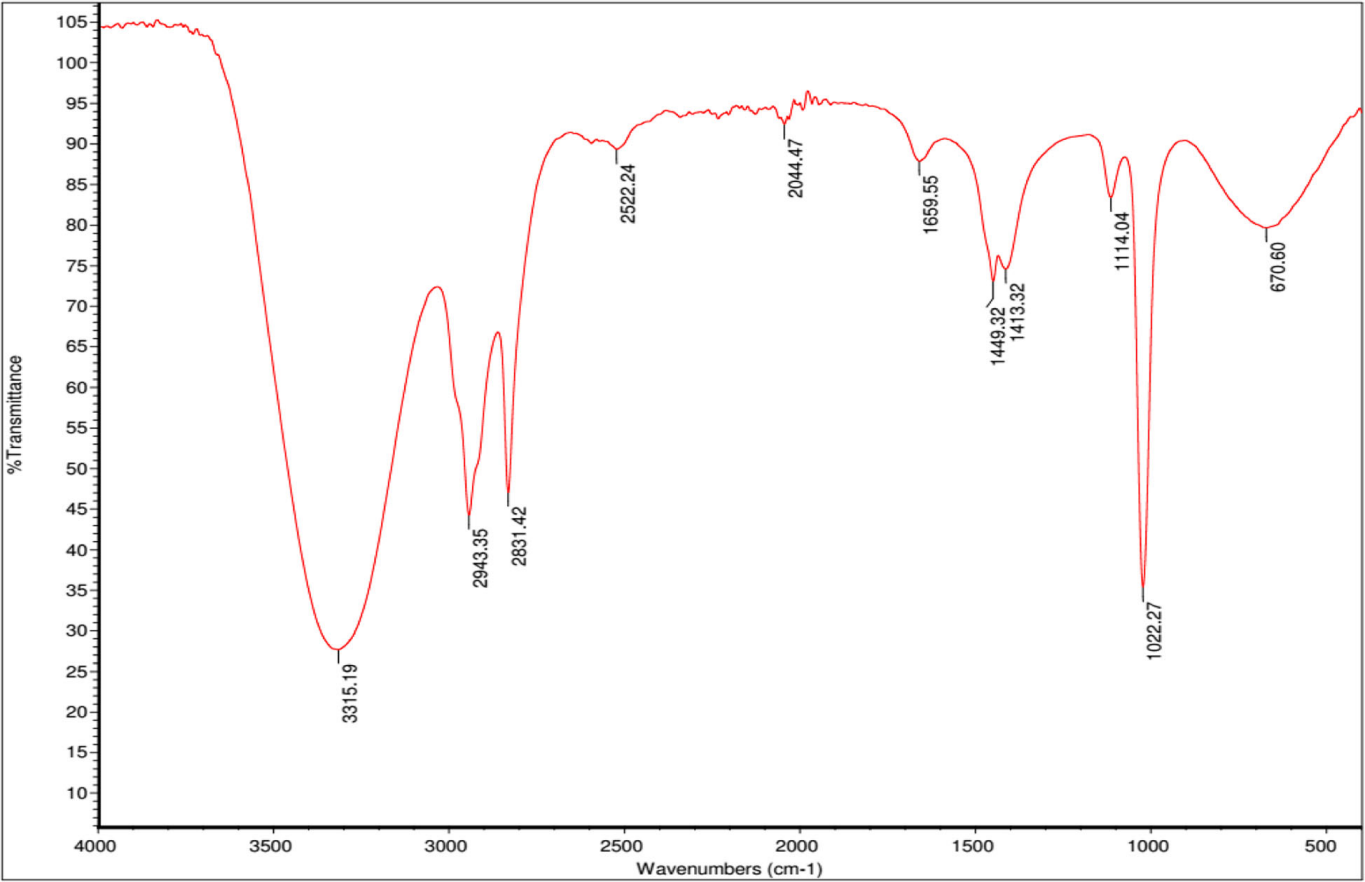
IR fingerprint of the rich-phenolic extract isolated from ethyl acetate fraction of *M. charantia* fruit extract

Fraction 2 was subjected to FTIR spectroscopy in order to identify toxic compound which caused cardiac toxicity in zebrafish embryos. According to the FTIR spectroscopy data; which is shown in Fig. 4, there are peaks occurring at 3315.19 cm-1 could be ascribed to the–OH stretching vibration of hydroxyl groups as well as hydrogen bonding. The band at around 2943.35 cm-1 and 2831.42 cm-1 was assigned to the asymmetric C–H stretching vibrations of methylene. The band located at 1022.27 cm-1 was due to C–O stretch vibration. The band at around 1449.32 cm-1 and 1413.32 cm-1 was due to the OH bending vibrations. The band located at 670.60 cm-1 attributed to O–H bending vibrations.

### The treatment of fruit extract of *M. charantia* perturbed the cardiac specification process during cardiac development in zebrafish embryos

**Fig. 5:**
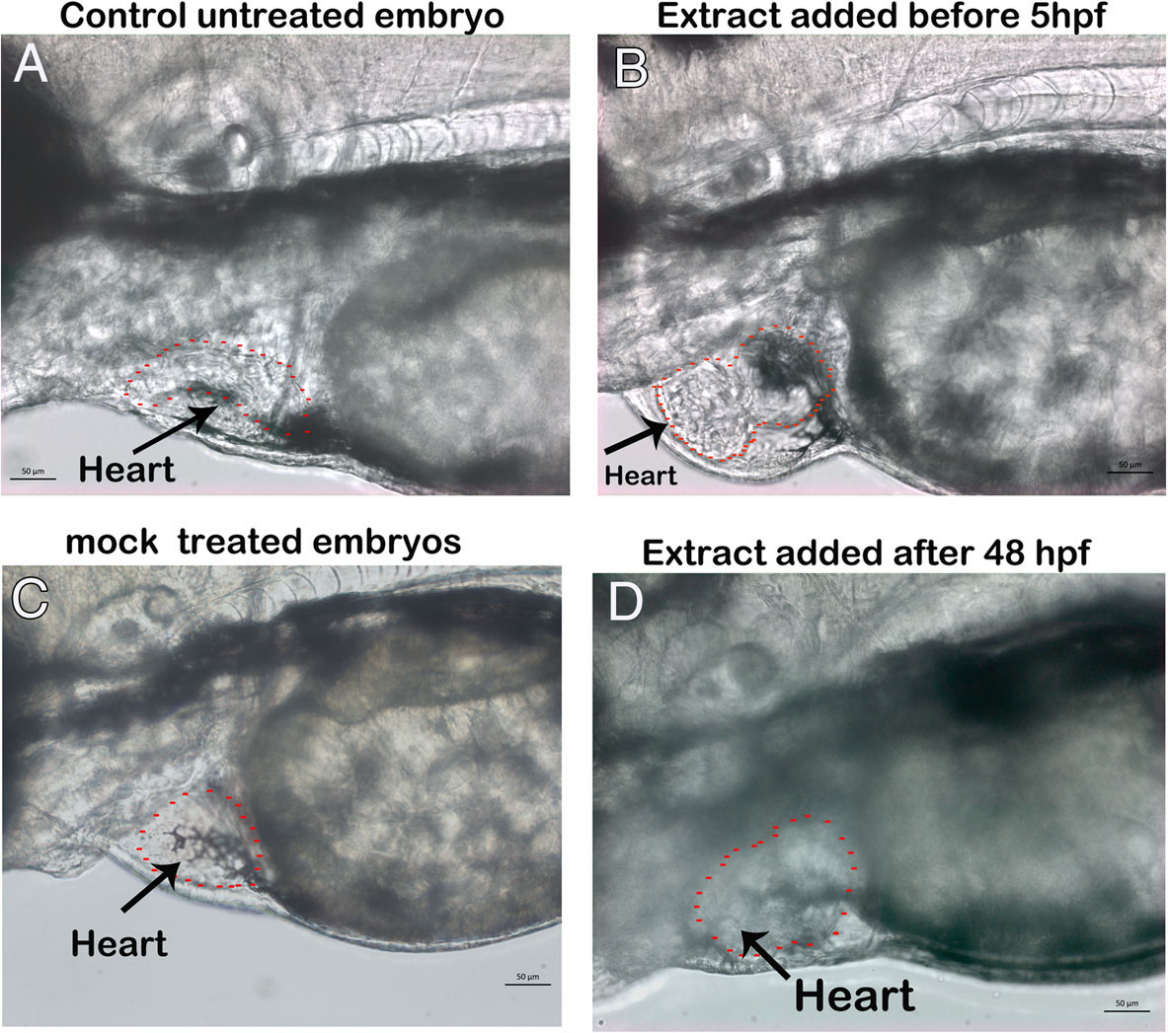
The fruit extract of *M .Charantia* effected the heart formation not the heart growth in treated zebrafish embryos. Representative live photomicrograph of zebrafish embryos at 72 hpf. **a**: Untreated control embryos at 72 hpf showing the normal formation and development of heart (red dots area represented by arrow). **b:** Zebrafish embryos which were treated with fruit extract of *M. charantia* at one cells stage exhibited the cardiac hypertrophy (red dots area and black arrow). **c:** The mock (methanol 0.5% v/v) treated embryos which were treated with solvent at one cell stage had normally heart development. **d:** Zebrafish embryos which were exposed to fruit extract of *M. charantia* at 48 hpf also had normal heart formation and development (red dot area). Abbreviation hpf: hours post fertilization. Scale bar 50 μm

The development or formation of any organ during embryonic development go through two distinct phases. Firs the stem cells are specified to develop into specific cell type and later these cells go through the process of differentiation. Once the cells are differentiated, the size of organ increases by rapid cell division. As it has been shown in this study that the crude fruit extract of *M. charantia* induced cardiac hypertrophy in zebrafish during the embryonic development. Moreover, the effect of crude fruit extract was much localized only heart and it did not disturbed the development of any other organ in zebrafish embryos. So the next question was to find out which process of heart development, (the specification and differentiation of cardiomyocytes or growth and expansion of heart) was altered by *M. charantia*. It could easily be determined if zebrafish embryos are exposed to crude fruit extract by following a time window treatment plan by exposing the zebrafish embryos to crude fruit extract at specified developmental stage of heart development. We planned a time window for exposing zebrafish embryos to the extract at two specific stages of cardiac development: i) before the onset of specification of myocardial progenitor cells (earlier than 5 hpf), ii) at 48 hpf, when heart has totally formed and become a functional organ. As shown in the Fig. 5a, e and f, the crude fruit extract was only active when it was added before the time of cardiac cell specification and differentiation. The cardiac hypertrophy was only observed in those zebrafish embryos which were treated with *M. charantia* before 5 hpf (cardiac cell specification stage) and exposure to *M. charantia* after this window, i.e. after 48 hpf, did not result in cardiac hypertrophy or any kind of observable malformation in zebrafish embryos.

**Fig. 6:**
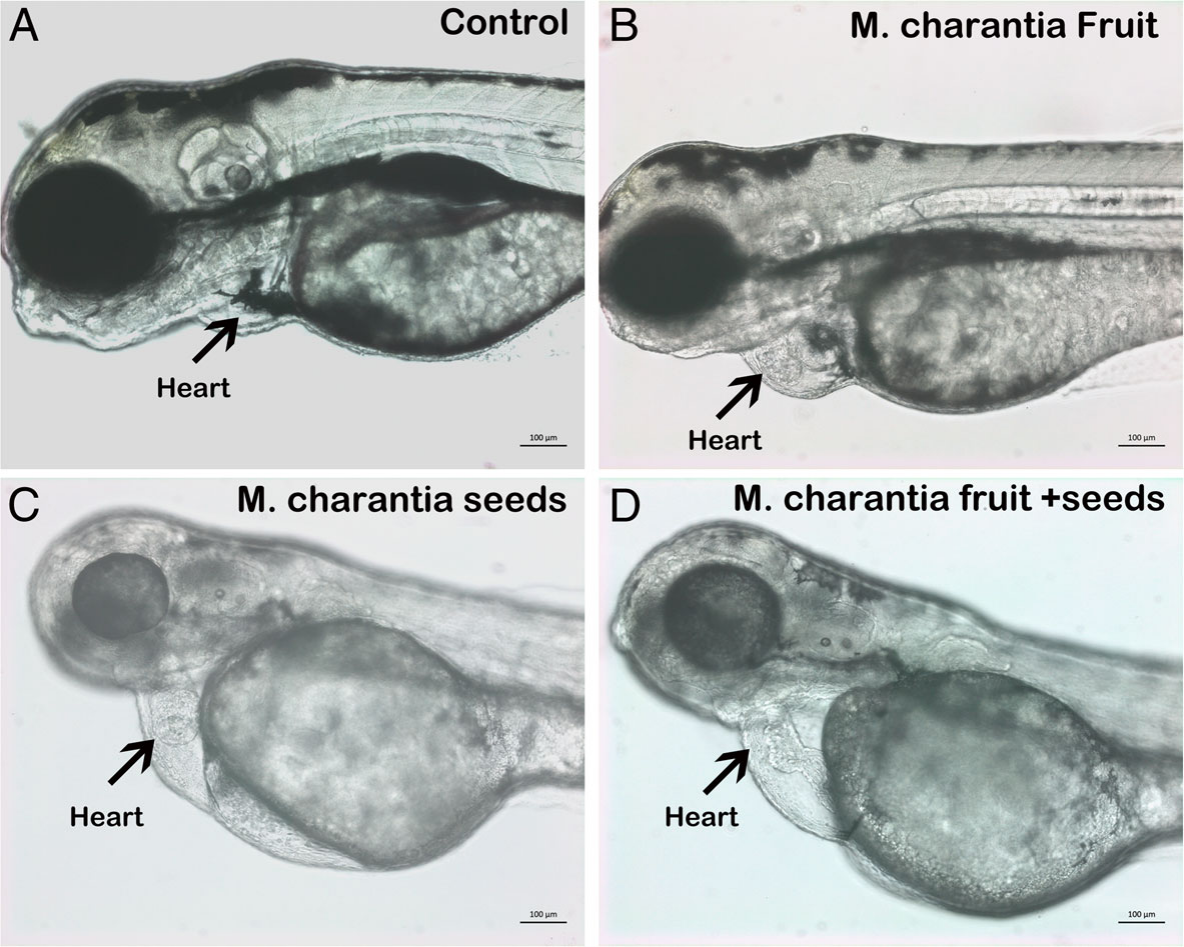
The co exposure of fruit and seeds extracts of *M. charantia* resulted lethality and severe cardiac toxicity in zebrafish embryos. Representative photomicrograph showing live zebrafish embryos at 3dpf control or treated with fruit, seeds or combination of seeds and fruit extract of *M. charantia*. **a** The mock (methanol 0.5%V/V) exposed embryos did develop normally and had normal heart growth and development. **b** The zebrafish embryos treated with fruit extract (100 μg/ml) had severe cardiac hypertrophy, **c** the embryos treated with 15 μg/ml of seeds extract had cardiac hypertrophy and also enlarged cardiac cavity. **d** The embryos which were treated with combination of both extract showed severe effect on heart formation as fused ventricle and atrium represented as string and also enlarged heart cavity

In order to find out the synergic effect or combined toxicity of seeds and fruit of *M. charantia* on zebrafish embryonic development, the zebrafish embryos were exposed to both extract at the same time. A combined dose of 50 μg/ml each of crude fruit and crude seeds extract, resulted 100% of lethality of embryos just after 24 h post treatment. Similarly, the combined doses in which crude seed extract was used at concentration of 30 μg/ml and crude fruit extract was used at concentration of 50 μg/ml also resulted 100% death of treated embryos. The combined dose monitoring of two extracts had shown that, the survival of embryos was only possible when the amount of crude seed extract was kept ≤5 μg/ml. As shown in Fig. 6, the embryos which were treated with 5 μg/ml of seeds extract and 30 μg/ml of fruit extract had survived up to 96hpf but exhibited severe cardiac malformation showing fusing of ventricle and atrium as string and had nonfunctional heart (no contraction and circulation).

## Discussion

The most common medicinal use of *M. charantia* is as an effective treatment of diabetes. Number of studies have been conducted to determine the safety profile of *M. charantia* in experimental animals; and all have found that *M. charantia* did not show any kind of toxicity when tested in non-pregnant animals [8, 51–56].

The safety profile of *M. charantia* is largely unknown in pregnant women so far, and consumption of *M. charantia* as an antidiabetic remedy by pregnant women cannot be overruled. Moreover, *M. charantia* has never been extensively studied in pregnant animals to elucidate the potential risk of *M. charantia* on fetus development. We have found only one report in the literature describing the teratological profile of *M. charantia* in pregnant experimental animals. Multiple congenital litter malformations were observed following exposure of pregnant Sprague Dawley rats to a water extract of fruit of *M. charantia* [57]. Guidelines by the Food and Drug Administration (FDA) clearly indicate that new developing drug that are intended for use in pregnant women must first be tested in suitable pregnant animal models [58].

The fruit and seeds of *M. charantia*, both are being used in anti-diabetic remedies [59, 60], however, the toxicity profile of fruit part in animal models systems has been investigated in previous reports but we did not find any published literature prior to this study, describing any toxicity profile of seeds of *M. charantia*. Various mammalian systems are traditionally used to examine the developmental toxicity of chemicals. The traditional methods are lengthy, costly, and often require a large number of animals, which raises ethical concerns. Moreover, testing any compound isolated from natural sources would be impractical owing to the limitation of quantity, because a large quantity of test material is normally needed for such screening methods. Zebrafish has emerged as a cost-effective and useful model for in vivo toxicity testing, and it is being routinely used for assessing the developmental toxicity of drugs or chemicals [43, 61]. Zebrafish embryos are very small in size; thus, by using this animal, it is possible to conduct a high-throughput screening in 90-well cell culture plates using very small quantity of test materials. Furthermore, a large number of embryos can be used for statistical application without ethical concerns because zebrafish embryo and larvae up to 5 dpf are exempted from requirement of approval by an ethical committee for animal use and care (Bartlett & Silk, 2016; Strahle et al., 2012).

The development process of zebrafish is largely comparable to that of mammalians [47, 62, 63], and this model is even used as a model for studying type 2 diabetes mellitus [64].

Treatment with crude seed extract of *M. charantia* to zebrafish embryos resulted in severely malformed embryos. The treated embryos were severely developmentally delayed than their non-treated counterparts, thus the seed extract or any supplementation prepared from the seed of *M. charantia* must be used with utmost care in pregnant women owing to the possible risk of fetus malformation, based on the result of this study.

The fruit part of *M.charantia* has been extensively studied and also lot of phytochemical has been identified from fruit part. The chemical constituents which have been identified from the fruit part of *M. charantia* has been best reviewed by [18]. The phytochemical from the seeds of *M. charantia* is largely unknown, hence only seeds extract was subjected to GC-MS analysis in this study. The GC-MS analysis helped to identify six new phytochemicals from the seeds of *M. charantia*. 1,2-CYCLOPENTANEDIONE has been identified with major peak which covered almost 60% of the areas. The molecular weight of,2-CYCLOPENTANEDIONE estimated to be 98.11. The biological activity of 1,2-CYCLOPENTANEDIONE is largely unknown but a similar compound 3-methyl-1,2-cyclopentanedione has been shown to be peroxisome proliferator-activated receptor γ (PPARγ) agonist [65]. Interestingly peroxisome proliferator-activated receptor γ (PPARγ) agonist are insulin sensitizers and widely used in the treatment of type 2 diabetes and cardiac hypertrophy has been reported in few preclinical studies using such agonists [66]. Whether 1,2-CYCLOPENTANEDIONE is PPARγ agonist or not need to be investigated experimentally, however, the cardiac hypertrophy in zebrafish embryos by seeds extract of *M. charantia* mimics the PPARγ agonist phenotype, which means 1,2-CYCLOPENTANEDIONE could be PPARγ agonist as well and hence 1,2-CYCLOPENTANEDIONE which has been identified as a major compound in seeds extract could be causing agent for cardiac hypertrophy in zebrafish embryos.

Treatment with the crude extract of unripe fruits of *M. charantia* did not induce sever level of malformation in zebrafish embryos in this study. The treated embryos were synchronous in development, as compared to the untreated control embryos. However, 100% of the treated zebrafish embryos developed cardiac hypertrophy, which was evident from 24 hpf onward. Severity of cardiac hypertrophy was directly correlated to the concentration of extract. The treated embryos developed cardiac hypertrophy after treatment with ≤25 μg/ml of fruit crude extract, whereas heart malformation was only evident in the zebrafish embryos treated with ≥30 μg/ml of fruit extract. The heart is the first organ to form and function during embryonic development in zebrafish, as in other vertebrates as well. Cardiac development in vertebrates usually begins with the specification of myocardial and endocardial progenitor cells, and cell-labeling techniques have helped visualizing the first myocardial progenitor cells in developing zebrafish embryos at approximately 5 hpf (before gastrulation starts) [67, 68]. The cardiovascular system of zebrafish is a closed system, as in other vertebrates, and the physiology of its cardiac cycle is highly representative of that of humans [69].

A specific time window treatment was planned to treat the embryos at different stages of cardiac development in order to elucidate either cardiac development or cardiac growth has been compromised by *M. charantia.*. Cardiac development monitoring during zebrafish embryonic development by various labeling and imaging techniques has revealed that the first myocardial progenitor cells undergo specification at approximately 5 hpf (before gastrulation starts) in developing zebrafish embryos. Myocardial cells start differentiation at the 12-somite stage (15 hpf). Cardiac looping takes place between 36 and 48 hpf, and functional heart, which has completed the cardiac development process, occurs at approximately 48 hpf [67, 68]. The cardiac hypertrophy was only apparent in treated zebrafish embryos when they were exposed to the crude extract of *M. charantia,* between one-cell and 5 hpf, that is before the onset of cardiac cell specification, and cardiac toxicity was not observed in those embryos which were treated with the same extract at same concentration at or after 48 hpf (after the cardiac development process had completed) and also did not show any obvious embryonic malformations. The exact mechanism and underlying molecular pathway which have been affected by *M. charantia* resulting the cardiac toxicity in zebrafish embryos is not known but the result from this study clearly indicates that the crude fruit extract of *M. charantia* affected the specification of myocardial cells and possibly by blocking those biological process and transcription factors which are normally required for the specification of myoblast. The cardiac toxicity of bitter gourd has also been reported in at least one another study. [58] Observed a significant increase in the cardiac weights of newly born litters of pregnant Sprague Dawley females rats which were treated with water fruit extract of *M. charantia* as compared to non-treated control group. The cardiac hypertrophy as observed in treated zebrafish embryos in this study confirmed the findings of [58] and hence the use of bitter gourd supplementation as anti-diabetic remedy in pregnant women must be use with extreme cautions to avoid any possible toxicity to developing fetus. The cardiac toxicity of *M. charantia* in adult animals or human is unknown; however, a clinical case of mild atrial fibrillation has been reported in one patient who consumed large amount of *M. charantia* juice [70].

## Conclusion

Herbs, as well as medicine or compounds derived from natural sources, have been generally anticipated to be free from toxicities and side effects, and the same was thought to be true for *M. charantia.* Cardiac toxicity induced by *M. charantia* fruit extract and gross teratological effects induced by *M. charantia* seed extract in zebrafish embryos in this study, as well as the results of the toxicity study in pregnant rats in a previous study, clearly warned that bitter gourd should be used with caution by pregnant women and that pharmacological studies should be conducted to determine the safe dose and duration of treatment with *M. charantia* in pregnant women.

## Additional files

Additional file 1: BG heart compressedR3. (MP4 2803 kb)

Additional file 2: BG seeds heartR3. (MP4 6156 kb)

Additional file 3: controlR3. (MP4 6156 kb)

## Data Availability

The raw data used and/or analyzed during the current study will be available from the corresponding author on reasonable request.

## Abbreviations

DPF: Days post fertilization
FTIR: Fourier-transform infrared spectroscopy
HPF: Hours post fertilization
HPLC: High-performance liquid chromatography
IACUC: An Institutional Animal Care and Use Committee
*M. charantia*: *Momordica charantia*
